# Osteoporosis Screening and Fracture Risk Assessment Tool: Its Scope and Role in General Clinical Practice

**DOI:** 10.7759/cureus.26518

**Published:** 2022-07-02

**Authors:** Sumant Chavda, Bharti Chavda, Rajani Dube

**Affiliations:** 1 Orthopedics, Ras Al Khaimah (RAK) Medical and Health Sciences University, Ras Al Khaimah, ARE; 2 General Surgery, Bristol Medical Center, Sharjah, ARE; 3 Obstetrics and Gynecology, Ras Al Khaimah (RAK) Medical and Health Sciences University, Ras Al Khaimah, ARE

**Keywords:** fracture risk assessment, fragility fractures, postmenopausal woman, screening tools, osteoporosis

## Abstract

Osteoporosis is a widely prevalent condition among postmenopausal women characterized by low bone mass and skeletal fragility that increases the risk of fractures specifically in the hip, spine, wrist, humerus, and pelvis. It has become a major public health problem around the world. An osteoporotic fracture affects one in every three women and one in every five men aged 50 and above. Hip and spine fractures are linked to a higher death rate and can cause ambulation problems, depression, chronic pain, independence loss, and persistent discomfort. It not only puts a lot of strain on the individual but also causes a significant cost to society. Osteoporosis is a silent disease that goes unrecognized until a patient develops a pathological fracture. Diagnosis of osteoporosis is based on bone mineral density (BMD) estimation by dual-energy x-ray absorptiometry (DXA) as defined by WHO. However, in many resource-constrained and underdeveloped or low-middle income countries, it is not widely available. There are a number of questionnaire-based techniques available to identify such postmenopausal women and older men who may be at risk of having low BMD and osteoporosis. Our aim of the study is to search and compile such simple yet useful and validated screening and assessment tools for osteoporosis that can help to identify people at risk of having low BMD and the potential candidate who can benefit from BMD estimation in a resource-restricted geographical area or low/middle-income countries and benefit from treatment. Though these tools are not diagnostic can have broader applicability in general clinical practice and usefulness in identifying high-risk individuals and may prove cost-effective. Although it has limitations, FRAX is a widely used osteoporotic fracture risk assessment tool around the globe and when used with femoral neck BMD it has greater accuracy.

## Introduction and background

Osteoporosis is a widespread condition marked by low bone mass, microarchitectural disturbance, and skeletal fragility, which increases the risk of fracture, especially in the hip, spine, wrist, humerus, and pelvis [1 ]. The process is gradual, painless, and often goes undiscovered earning it the moniker a “silent disease". According to the World Health Organization’s (WHO) categorization, BMD evaluation by DXA is the standard test to diagnose osteoporosis. The BMD diagnostic thresholds (by DXA) were defined by WHO based on the SD difference between a patient's BMD and that of a young adult reference population (T-score) and is shown in Table 1. 

**Table 1:** WHO definition of osteoporosis based on BMD SD- Standard Deviation

Classification	BMD	T-Score
Normal	Within 1 SD of the mean level for a young-adult reference population	T-score at −1.0 and above
Low bone mass (osteopenia)	Between 1.0 and 2.5 SD below that of the mean level for a young-adult reference population	T-score between −1.0 and −2.5
Osteoporosis	2.5 SD or more below that of the mean level for a young-adult reference population	T-score at or below −2.5
Severe or established osteoporosis	2.5 SD or more below that of the mean level for a young-adult reference population with fractures	T-score at or below −2.5 with one or more fractures

Osteoporosis has become a major public health problem around the world. An osteoporotic fracture affects one in every three women and one in every five men aged 50 and above. A higher risk of osteoporosis-related fracture has been linked to a variety of conditions. Lifestyle factors, hereditary diseases, hypogonadal states, endocrine disorders, gastrointestinal disorders, hematologic disorders, rheumatologic and autoimmune diseases, neurological and musculoskeletal risk factors, medicines, and other conditions and diseases fall under this category and are shown in Table 2 [2].

**Table 2:** Clinical risk factors for osteoporosis

Life style factors		
Alcohol abuse	High salt intake	Low calcium intake
Excessive thinness	Immobilization	Smoking (active or passive)
Excess vitamin A	Inadequate physical activity	Vitamin D insufficiency
Frequent falling		
Genetic disease		
Cystic fibrosis	Hypophosphatasia	Osteogenesis imperfecta
Ehlers-Danlos	Hypophosphatemia	Parental history of hip fracture
Gaucher’s disease	Marfan syndrome	Porphyria
Hemochromatosis	Menkes steely hair syndrome	
Hypogonadal states		
Anorexia nervosa	Hyperprolactinemia	Premature menopause (<40 yrs.)
Androgen insensitivity	Hypogonadism	Turner’s & Klinefelter’s syndromes
Athletic amenorrhea	Panhypopituitarism	
Endocrine disorders		
Central obesity	Diabetes mellitus (Types 1 & 2)	Thyrotoxicosis
Cushing’s syndrome	Hyperparathyroidism	
Gastrointestinal disorders		
Celiac disease	Gastrointestinal surgery	Pancreatic disease
Bariatric surgery	Inflammatory bowel disease	Primary biliary cirrhosis
Gastric bypass	Malabsorption syndromes	
Hematologic		
Hemophilia	Multiple myeloma	Systemic mastocytosis
Leukemia and lymphomas	Sickle cell disease	Thalassemia
Monoclonal gammopathies		
Rheumatologic and autoimmune diseases		
Ankylosing spondylitis	Other rheumatic and autoimmune diseases	Systemic lupus
Rheumatoid arthritis		
Neurological and musculoskeletal diseases		
Epilepsy	Multiple sclerosis	Spinal cord injury
Muscular dystrophy	Parkinson’s disease	Stroke
Medications		
Aluminum-containing antacids	Depo-medroxyprogesterone (premenopausal contraception)	Parental nutrition
Androgen deprivation therapy	Glucocorticoids (≥ 5 mg/d prednisone or equivalent for ≥ 3 months)	Proton pump inhibitors Selective serotonin reuptake inhibitors
Anticoagulants (heparin)	Anticonvulsants	Aromatase inhibitors
Thyroid replacement hormone (in excess)	GnRH (Gonadotropin releasing hormone) agonists	Tamoxifen® (premenopausal use)
Barbiturates	Cancer chemotherapeutic drugs	Methotrexate
Thiazolidinediones (such as Actos® and Avandia®)	Lithium Cyclosporine A and tacrolimus	
Miscellaneous conditions and diseases		
HIV/AIDS	Congestive heart failure	Idiopathic scoliosis
Amyloidosis	Depression	Post-transplant bone disease
Chronic metabolic acidosis	End stage renal disease	Sarcoidosis
Chronic obstructive lung disease	Hypercalciuria	Weight loss

Because of the porosity, osteoporosis makes bones weaker, resulting in osteoporotic fractures (fragility fractures/low-energy fractures). Low-energy fractures are those that occur as a result of a fall from a standing height or less, without a substantial trauma such as a car collision. In the remaining years of their lives, approximately 50% of postmenopausal women and 20% of males over the age of 50 will suffer a fragility fracture [3]. In the year 2000, nine million osteoporotic fractures were reported worldwide, with 1.6 million hip fractures, 1.7 million forearm fractures and 1.4 million clinical vertebral fractures [4]. Hip and spine fractures are linked to a 10%-20% higher death rate [1,5]. Fractures can cause ambulation problems, despair, chronic pain, loss of independence, and persistent discomfort [6,7]. These fragility fractures put a lot of strain on the individual and add significant costs to society [8,9]. Medicare currently pays for approximately 80% of these fractures, with hip fractures accounting for 72% of fracture costs. Due to an aging population, the cost of care is expected to rise to $25.3 billion by 2025 in the USA [10].

The majority of men and women who are at high risk of fracture are not diagnosed or treated, and multiple studies have found that case-finding procedures and strategies used in many countries are ineffective [11,12]. Screening for osteoporosis among the general population using the DXA scan alone may not be feasible, as this test is expensive, and is only available in resource-rich hospitals. This has resulted in the development of several osteoporosis risk assessment tools. These techniques combine risk factors such as age, low body weight, history of fractures, and glucocorticoid usage into a single assessment of fracture risk for an individual. These tools are designed to either identify people who are at a higher risk of fractures (with the option to include a BMD result in the risk scoring) or people who are at a higher risk of having low BMD. Because the effect of BMD on fracture risk is modified by the presence of clinical risk factors, fracture risk assessment tools have also been used to help doctors decide whether or not to refer patients for a BMD measurement [13]. Assessment of bone mineral density (BMD) by the dual-energy x-ray absorptiometry (DXA) is the gold standard in the diagnosis of osteoporosis [14]. However, in many resource-constrained and underdeveloped countries, it is not widely available [15]. As a result, other risk assessment tools that are available are used to identify postmenopausal women and older men who are at high risk for osteoporosis.

## Review

Aim and objective

Our aim and objective of the study are to search and compile the simple yet useful and validated screening and assessment tools for osteoporosis that can help to identify people at risk of having low BMD and the potential candidate who can benefit from BMD estimation in a resource-restricted geographical area or in low-middle income countries.

Methodology

We conducted a thorough review of publications concentrating on screening techniques for osteoporosis diagnosis. To prepare this narrative review, the search engines, PubMed and Google Scholar were used to find original and review publications written in the English language using the terms “postmenopausal women,” “osteoporosis,” “risk assessment tools,” “screening tools,” and “fragility fractures.” The article published in languages other than English were not searched. 

Discussion

To calculate the risk of osteoporosis the screening techniques include simple questionnaires that measure a composite of risk variables such as advanced age, high‑risk ethnic group, weight, glucocorticoid usage, or hormone replacement therapy. Thereby these techniques overcome the disadvantages of high costs and lack of equipment availability to assess the risk of low BMD [16,17]. The body weight (WEIGHT), The Simple Calculated Osteoporosis Risk Estimation (SCORE), the Age, Bulk, One or Never Estrogen (ABONE), the Osteoporosis Risk Assessment Instrument (ORAI), the Osteoporosis Self-assessment Tool for Asians (OSTA), the Instrument and Osteoporosis Index of Risk (OSIRIS), the Osteoporosis Prescreening Risk Assessment (OPERA) and the Malaysian Osteoporosis Screening Tool (MOST) are some of the few tools available for identifying women possibly having low BMD and osteoporosis. The Male Osteoporosis Risk Estimation Score (MORES) is an osteoporosis screening tool designed to identify males who are at risk of developing the disease. Estimation of fracture risk can be done using Fracture Risk Assessment Tools (FRAX) developed at the University of Sheffield, and some others, such as the Garvan fracture risk calculator and the Qfracture scores. 

Development and validation of osteoporosis screening tools

WEIGHT (1996) was developed by determining the relationships between body measure (weight, height, body mass index, lean tissue mass, fat mass, waist-to-hip ratio) and bone mineral density (BMD) in 175 women of ages 28-74 years in a cross-sectional study in a county in central Sweden [18]. It was found by using multivariate logistic regression models that weight of over 71 kg was associated with a very low risk of being osteopenic compared with women weighing less than 64 kg. Furthermore, a sensitivity/specificity analysis revealed that, in this population, a woman weighing over 70 kg is not likely to have osteoporosis with sensitivity of 94% and specificity of 36% [18]. This tool is also have been validated in Malaysia and Singapore [19-21].

SCORE (1998) was developed in USA in a cohort of 1424 postmenopausal women using only the six parameters of age, weight, race, the presence of rheumatoid arthritis (RA), history of fractures and the use of estrogen therapy [22,23]. A total 106 investigators specializing in family medicine, geriatrics, internal medicine, endocrinology, rheumatology or gynecology participated in the study. Each woman in the cohort was asked to complete a self-administered questionnaire composed of approximately 60 questions on factors possibly or probably associated with osteoporosis covering demographics, body measurements, lifestyle data, reproductive history, other medical history and current or past medication. A simple additive scoring system was developed by using regression modelling to identify factors most predictive of low bone density at femoral neck. The sensitivity of 89% and specificity of 50% was achieved during validation in another group. This tool is validated in Belgium, Netherland, Japan and Singapore as well [20,24-26].

ABONE (2000) was developed by administering questionnaire to 1,610 participating postmenopausal women in USA. Data analysis was performed by Chi test and multivariate logistic regression and simple scoring system was developed by using three variable age, weight, and estrogen therapy [27]. This tool was also validated in Singapore [20].

ORAI (2000) was developed and validated using Ontario baseline data from the Canadian Multicenter Osteoporosis Study. The study population comprised 1,376 women, of whom 926 were allocated to the development of the tool and 450 to its validation. A simple algorithm based on age, weight and current estrogen use (yes or no) was developed [28]. Osteoporosis Risk Assessment Instrument (ORAI) showed that the tool had a sensitivity of 93.3% (95% confidence interval [CI] 86.3%-97.0%) and a specificity of 46.4% (95% CI 41.0%-51.8%) for selecting women with low bone mineral density [28]. Use of the ORAI represented a 38.7% reduction in DXA testing compared with screening all women in this study [28]. This tool also is been validated in USA, Belgium, Netherland, Japan and Singapore [20,24-26].

OSTA (2001) was developed using information collected by administering questionnaire in 860 postmenopausal Asian women in eight countries (China, Taiwan, Hong Kong, Korea, Malaysia, Singapore, Thailand, and the Philippines) and validated by using it on a sample of postmenopausal Japanese women [26,29]. OSTA was calculated as one‑fifth of the difference between weight in kilogram and age in years [29]. OSTA has a sensitivity of 91% and specificity of 45%. This tool has also been validated in USA, Canada, Belgium, Netherland, Korea, Thailand, Hong Kong and Singapore [24,25,30-33].

OSIRIS (2002) was designed based on an extensive review of the literature evaluating risk factors for osteoporosis, and tested its performance in a large cohort of 1303 postmenopausal women in whom BMD was measured by dual x-ray absorptiometry. The Osteoporosis Index of Risk (OSIRIS) is based on four variables: age, body weight, current hormone replacement therapy uses, and history of previous low impact fracture. The sensitivity and specificity for an OSIRIS value of +1 were respectively 78.5% and 51.4%. Three categories were arbitrarily created using OSIRIS, with under cut-off of +1 and -3. The low-risk category (OSIRIS > +1) represented 41% of all women; only 7% of the women in this category had osteoporosis. The prevalence of osteoporosis was very high (66%) among the group at high risk (OSIRIS < -3 representing 15% of all women). The prevalence of osteoporosis was 39% in the intermediate risk group (-3 < OSIRIS < +1, 44% of all women) [34].

OPERA (2004) was developed by analyzing the records of 1,522 postmenopausal Italian females over 50 years of age who had undergone testing with DXA35. Osteoporosis risk index scores were compared to bone density T-scores. A simple algorithm based on age, weight, history of previous low impact fracture, early menopause, and corticosteroid therapy was developed. Validation of this five-item osteoporosis prescreening risk assessment (OPERA) index showed that the tool, at the recommended threshold (or cutoff value) of two, had a sensitivity that ranged from 88.1 (95% confidence interval [CI] for the mean: 86.2%-91.9%) at the femoral neck to 90% (95% CI for the mean: 86.1%-93.1%) at the lumbar spine area. Corresponding specificity values were 60.6 (95% CI for the mean: 57.9%-63.3%) and 64.2% (95% CI for the mean: 61.4%-66.9%), respectively [35].

MORES (2007) was developed and validated by using risk factor data from the National Health and Nutrition Examination Survey III to develop a best fitting multivariable logistic regression model in men aged 50 years and older randomized to either the development (n = 1,497) or validation (n = 1,498) cohorts [36]. The best fitting model was transformed into a simplified scoring algorithm, the MORES. The MORES included three variables - age, weight, and history of chronic obstructive pulmonary disease - and showed excellent predictive validity in the validation cohort [36]. A score of 6 or greater yielded an overall sensitivity of 0.93 (95% CI, 0.85-0.97), a specificity of 0.59 (95% CI, 0.56-0.62). MORES identifies men at higher risk of osteoporosis (cut-off ≥6) who should undergo a diagnostic DXA scan [36].

MOST (2010) was developed in Malaysia by assessing the correlation between clinical risk factors and low BMD among 586 healthy women aged 45 years and above. They developed a simple additive scoring system utilizing parameter of age, years of menopause, BMI and hip circumference. A score of ≥ 4, the screening tool had a sensitivity of 73.2%, a specificity of 61.6% for identifying women with low BMD (T score ≤ -2) plus a sensitivity of 80.2% in selecting women with osteoporosis [19]. The parameters utilized in these tools and the cutoffs used to identify postmenopausal women and elderly men at risk for osteoporosis are shown in Table 3.

**Table 3:** List of osteoporosis screening tools with cut-offs and parameter used WEIGHT- The body weight, MOST -the Malaysian Osteoporosis Screening Tool, SCORE- The Simple Calculated Osteoporosis Risk Estimation (SCORE), ABONE- the Age, Bulk, One or Never Estrogen, ORAI- the Osteoporosis Risk Assessment Instrument, OSTA- the Osteoporosis Self-assessment Tool for Asians, OSIRIS- the Instrument and Osteoporosis Index of Risk, OPERA- the Osteoporosis Prescreening Risk Assessment, RA- Rheumatoid arthritis, COPD- Chronic obstructive pulmonary disease, BMI – Body mass index, kg – Kilogram, cm – centimeter

Screening Tool	Cut off Point	Risk factors	Score	Conditions
WEIGHT [18]	70 kg	Weight		Weight of ≤ 70 kg
MOST [19]	≥ 4	Age (Years) > 61	20	
		56-60	06	
		51-55	02	
		< 50	00	
		Years of post-menopause > 10	22	
		6-10	06	
		1-5	04	
		00	00	
		BMI <19 kg/m^2^	04	
		19-24 kg/m^2^	02	
		> 24 kg/m^2^	00	
		Hip circumference		
		< 90 cm	02	
		> 90 cm	00	
SCORE [23]	≥ 6	Race	+5	Woman is not black
		RA	+4	Woman has Rheumatoid arthritis
		History of fractures	+4	For each type (wrist, rib, hip) of nontraumatic fracture after age 45 (maximum=12)
		Age (years)	+3	Times first digit of age in years
		Estrogen therapy	+1	Woman has never received estrogen therapy
		Weight	-1	Times weight in pounds divided by 10 and truncated to nearest integer
ABONE [27]	≥ 2	Age (years) >65	01	
		Weight (kg) <63.5	01	
		Estrogen therapy	01	Woman has never received estrogen therapy
ORAI [28]	≥ 9	Age (years) >75	15	
		65-74	09	
		55-64	05	
		45-54	00	
		Estrogen therapy	02	Woman has never received estrogen therapy
		Weight (kg) <60	09	
		60-69	03	
		≥70	00	
OSTA [29]	≤ -1	Age (years)		0.2× (body weight [kg] − age [years])
		Weight (kg)		
OSIRIS [34]	+1 and – 3	Age (years) x -2		Remove last digit
		Weight (kg) x 2		Remove last digit
		Estrogen therapy	+2	Woman has never received estrogen therapy
			-2	History of low impact fracture
		Categories	> +1	Low risk
			< -3	High risk
			-3< OSIRIS	Intermediate risk
OPERA [35]	≥ 2	Age (years) ≥65	01	
		Weight (kg) <57	01	
		History of fractures	01	Low trauma fracture after age of 45
		Menopause	01	Early before age of 45 years
		Steroid use	01	> 5 mg/day for > 6 months
MORE [36]	≥ 6	Age (years) ≤55	00	
		56-74	03	
		≥ 75	04	
		Body Weight (kg)		
		≤ 70	06	
		71-80	04	
		> 80	00	
		COPD yes	03	Chronic obstructive pulmonary disease
		No	00	

All of these tools perform somewhat similarly and forecast with a moderate level of accuracy. Toh et al. compared and assessed the performance of six osteoporosis risk assessment tools (SCORE, ORAI, ABONE, BMOS, MOST, OSTA) for screening osteoporosis in Malaysian postmenopausal women and concluded that all six risk assessment tools performed equally and well in terms of sensitivity while moderate in terms of specificity [37]. In another prospective study by Rubin et al. in Denmark comparing FRAX, OST, ORAI, OSIRIS and SCORE concluded that FRAX did not perform better than the other simpler tools and it would be easier to use simpler tools by the GP or patient herself in clinical practice [38]. In one Egyptian study comparing seven osteoporosis screening tools among 681 elderly women by Abou-Hashem et al. it was opined that among all seven screening tools performed well, however SCORE performed better than other [39]. In one population based Canadian study to inform practice guidelines in Canada, Leslie et al comparing SCORE, ORAI, SOFSURF, OSIRIS, ABONE, OST and FRAX-MOF concluded that all screening tools show some ability to identify individuals qualifying for treatment and stratify risk for incident fracture, however best performing strategy was FRAX-MOF without BMD using cut-off of ≥ 10% [40]. Table 4 shows the relative sensitivity and specificity of some of these screening models.

**Table 4:** Sensitivity and specificity of osteoporosis screening tools

Screening model	Sensitivity	Specificity	Number of participants
WEIGHT [18]	94	36	175
MOST [19]	80	62	586
SCORE [23]	89	50	1426
ABONE [27]	NA	NA	1610
ORAI [28]	93	46	1376
OSTA [29]	91	45	860
OSIRIS [34]	79	51	1303
OPERA [35]	88	61	1522
MORE [36]	93	59	2995

Development and validation of fracture risk assessment tools

Fracture Risk Assessment Tool (FRAX)

**Figure 1:**
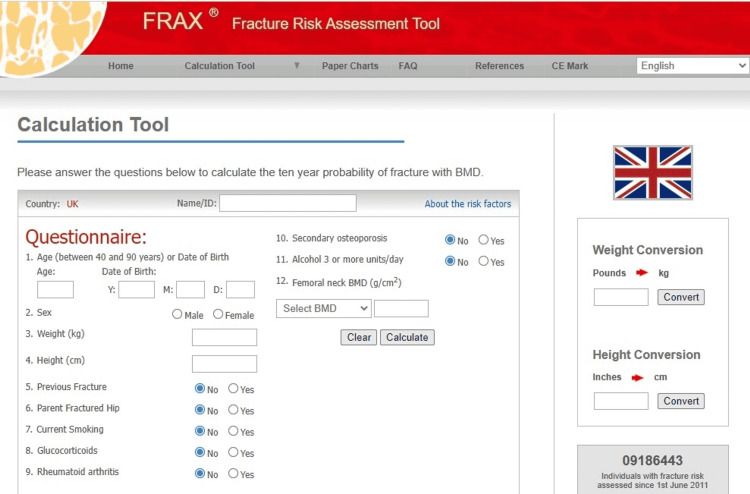
Screen page of FRAX calculator (UK model) adapted from web http://www.shef.ac.uk/FRAX) [41-43]

FRAX was launched in 2008 by the University of Sheffield which calculates the risk of 10-year probability of hip fracture and major osteoporotic fracture (hip, spine, proximal humerus, or forearm) for untreated patients aged 40 to 90 years using easily obtainable clinical risk factors for fracture and femoral neck BMD (g/cm, using dual-energy x-ray absorptiometry [DXA]), when available [41]. The clinical risk factors included in calculation are age, gender, weight, height, previous fracture, parent fractured hip, current smoking, glucocorticoids, rheumatoid arthritis, secondary osteoporosis and alcohol 3 or more units per day. FRAX is based on data collected from large, prospective, observational studies in which clinical risk factors, BMD and fractures of females and males of different ethnicities and from different world regions were evaluated [42,43]. FRAX has been validated in about 26 independent cohorts, with the majority of the participant being women [44]. The statistical power of this huge dataset allows for the estimation of fracture probability from a set of risk factors specific to an individual. The country-specific FRAX prediction algorithms are available for many countries online (Figure 1). The FRAX calculator is also available on current versions of DXA software and as an app for smartphones [45].

FRAX is a useful clinical tool for determining the risk of fracture. However, there are obvious limitations like with any clinical tools. Lack of extensive validation in treated patients, limitation to four ethnic groups in the United States (White, Black, Hispanic, and Asian Americans), uncertainty about the range of error with fracture risk, and lack of validation with BMD measurements by technologies other than DXA are just a few of the drawbacks [46]. In untreated patients, the FRAX algorithm calculates fracture probability using femoral neck BMD (g/cm). BMD input from non-hip sites and other hip regions of interest has not been validated with FRAX and is therefore not recommended [41].

Other fracture risk assessment models are available (e.g., QFracture, Garvan), but most have not been validated in diverse populations, and they are not in widespread use [38,44,47]. The thresholds for intervention vary with the individual models. The selection of a particular assessment tool may best be determined by country-specific guidelines for treatment thresholds.

Vertebral fracture assessment (VFA)

Even in absence of BMD diagnosis, a presence of a vertebral fracture is consistent with a diagnosis of osteoporosis [48]. The majority of spinal fractures are asymptomatic when they first occur and often are undiagnosed for many years. The only way to diagnose these fractures is via proactive vertebral imaging. The discovery of a previously unrecognized vertebral fracture may change the diagnostic categorization, future fracture risk assessment and treatment decisions [49]. Radiographically confirmed vertebral fractures (even if fully asymptomatic) are a marker of reduced bone quality and strength and a powerful predictor of new vertebral and other fractures, regardless of BMD, age, and other clinical risk factors. The presence of a single vertebral fracture increases the risk of subsequent fractures fivefold and the risk of hip and other fractures two- to threefold [50]. A lateral thoracic and lumbar spine x-ray, which is accessible on most modern DXA equipment or x-ray machines can be used for vertebral imaging. VFA can be conveniently performed at the time of BMD assessment, while conventional x-ray may require referral to a standard x-ray facility.

Because vertebral fractures are so prevalent in older individuals and most fractures produce no acute symptoms, National Osteoporosis Foundation (NOF) recommends vertebral imaging tests in individuals as defined in Table 5. 

**Table 5:** Indications for vertebral imaging SN- serial number

SN	Indications
1	All women age 70 and older and all men age 80 and older if BMD T-score at the spine, total hip, or femoral neck is ≤−1.0
2	Women age 65 to 69 and men age 70 to 79 if BMD T-score at the spine, total hip, or femoral neck is ≤−1.5
3	Postmenopausal women and men age 50 and older with specific risk factors: Low-trauma fracture during adulthood (age 50 and older) Historical height loss of 1.5 in. or more (4 cm) Prospective height loss of 0.8 in. or more (2 cm) Recent or ongoing long-term glucocorticoid treatment

Once a first vertebral imaging test is done, it only needs to be repeated if prospective height loss is documented or new back pain or postural change occurs [51,52].

## Conclusions

Osteoporosis screening tools are not diagnostic tools as they do not include BMD assessment by DXA, which is a gold standard test for diagnosing osteoporosis as defined by WHO. These tools are designed to predict low BMD and thereby indirectly probability of low-energy fractures. They are simple and validated tools that can be used by GP in clinical practice and may have broader applicability and usefulness in identifying at-risk individuals in resource-restricted and low‑ to middle‑income countries where the availability of DXA scanners is limited and unaffordable for many and may prove cost-effective. Although it has limitations, FRAX is a widely used osteoporotic fracture risk assessment tool around the globe and when used with femoral neck BMD it has greater accuracy.
